# Elevated neutrophil-to-lymphocyte ratio and the incidence of autoimmune diseases: evidence from a large prospective cohort study

**DOI:** 10.1038/s41598-025-21188-y

**Published:** 2026-01-06

**Authors:** Dongwon Yoon, Choa Yun, Isabel Beerman, May A. Beydoun, Lenore J. Launer, Minkyo Song

**Affiliations:** 1 Laboratory of Epidemiology & Population Sciences, National Institute on Aging, National Institutes of Health, 251 Bayview Blvd, Baltimore, MD 21224 USA; 2 Translational Gerontology, National Institute on Aging, National Institutes of Health, Baltimore, USA; 3 College of Pharmacy, Ewha Womans University, Seoul, Korea

**Keywords:** Neutrophil, Lymphocyte, Neutrophil-to-lymphocyte ratio, Autoimmune disease, Prospective cohort, UK biobank, Epidemiology, Biomarkers, Rheumatic diseases, Autoimmune diseases

## Abstract

We prospectively assessed the association between neutrophil-to-lymphocyte ratio (NLR) and incident autoimmune disease risk using data from 430,347 UK Biobank participants recruited between 2006 and 2010. The NLR was calculated by dividing neutrophil counts by lymphocyte counts and categorized into quartiles. Cox regression models estimated hazard ratios (HRs) for per quartile increase of (1) a composite of 39 types of autoimmune diseases, (2) each specific autoimmune diseases, applying a 2-year lag-period and Bonferroni adjustments. Natural cubic spline analysis was performed to assess the threshold of NLR associated with autoimmune diseases. Among 430,347 participants (mean age: 56.4 years, 53.9% female), elevated NLR was significantly associated with any autoimmune diseases (27,571 events, HR per quartile increase: 1.09, 95% CI: 1.08–1.10; P-trend < 0.01). A non-linear association was observed, with risk significantly increasing above an NLR threshold of 2.51. Participants in the highest quartile (Q4) had a higher risk compared to Q1 (HR 1.30, 95% CI 1.26–1.35). Of 39 autoimmune diseases, 14 showed significant associations, most notably sarcoidosis (HR 1.52, 95% CI 1.38–1.67), antiphospholipid syndrome (1.46, 1.26–1.69), and autoimmune hepatitis (1.37, 1.20–1.56). These findings suggest elevated NLR as a potential pre-clinical indicator for identifying individuals at increased risk of autoimmune diseases.

**Supplementary Information:**

The online version contains supplementary material available at 10.1038/s41598-025-21188-y.

## Introduction

 Neutrophils have been recognized as the effector cells in acute inflammatory responses, rapidly migrating to infection or injury sites and releasing cytokines, antimicrobial peptides, and reactive oxygen species to eliminate pathogens^1^. In contrast, lymphocytes are essential components of adaptive immunity^2^, which regulate immune responses by producing antibodies, modulating inflammatory process, and ensuring immune tolerance. However, dysregulation of either or both neutrophil and lymphocyte function, such as excessive activation or impaired regulatory mechanisms, can contribute to chronic inflammation, potentially leading to autoimmune disease^3,4^.

The neutrophil-to-lymphocyte ratio (NLR) has emerged as a valuable marker of systemic inflammation, reflecting the balance between innate and adaptive immunity^5^. In healthy adults, a normal NLR typically ranges from 1 to 2, while values above 3.0 or below 0.7 generally indicate a pathological inflammatory state^6^. This imbalance has been linked to various chronic conditions, including cardiovascular diseases^7^, cancer^8^, and metabolic disorders^9^. In the context of autoimmune diseases, an elevated NLR may serve as an early indicator of immune activation and disease progression, highlighting its potential role as a biomarker for assessing disease risk.

Given the growing evidence of the role of systemic inflammation in chronic disorders, NLR has gained attention as a simple, cost-effective biomarker for early risk evaluation. However, current evidence regarding the association between NLR and autoimmune disease risk remains limited by cross-sectional associations and small sample sizes and high-heterogeneity across different populations and conditions^10–15^. Therefore, we aimed to investigate the association between NLR and incident autoimmune diseases in a large-scale prospective cohort.

## Results

### Characteristics of study population

The mean age at recruitment was 56.4 years (SD 8.1), and 53.9% were female (Table 1). Age distribution remained consistent across quartiles, but the proportion of females decreased from 59.5% (Q1) to 46.7% (Q4). While socioeconomic characteristics were generally similar across quartiles, there were slight differences in health behavior variables. The highest NLR quartile showed a greater proportion of current smokers (11.4% in Q4 vs. 9.6% in Q1) and current alcohol consumers (92.3% vs. 91.5%). Similarly, the prevalence of comorbidities increased progressively across quartiles, with the highest rates in Q4; cardiovascular diseases (6.5% in Q4 vs.4.0% in Q1), hypertension (29.2% vs. 23.2%), dyslipidemia (15.6% vs. 13.5%), diabetes (5.2% vs. 4.1%), and cancer (10.0% vs. 8.5%).

**Table 1:** Baseline characteristics of the study population for overall and quartiles of neutrophil-to-lymphocyte ratio in UK biobank.

	Overall (*N* = 430,347)	Q1^*^ (*N* = 107,656)	Q2^*^ (*N* = 107,518)	Q3^*^ (*N* = 107,589)	Q4^*^ (*N* = 107,584)
Demographic, 2006–2010					
Age at recruitment years, mean (SD)	56.4 (8.1)	56.2 (7.8)	56.3 (8.0)	56.3 (8.2)	56.7 (8.4)
Female sex	232,103 (53.9)	64,109 (59.5)	60,652 (56.4)	57,126 (53.1)	50,216 (46.7)
Race/Ethnicity					
White	405,706 (94.3)	96,759 (89.9)	101,866 (94.7)	103,110 (95.8)	103,971 (96.6)
Asian	9,669 (2.2)	3,619 (3.4)	2,519 (2.3)	2,019 (1.9)	1,512 (1.4)
Black	6,693 (1.6)	4,377 (4.1)	1,149 (1.1)	689 (0.6)	478 (0.4)
Mixed	2,525 (0.6)	720 (0.7)	655 (0.6)	593 (0.6)	557 (0.5)
Other	5,754 (1.3)	2,181 (2.0)	1,329 (1.2)	1,178 (1.1)	1,066 (1.0)
**Socioeconomic**					
Income					
Less than £18,000	80,525 (18.7)	18,476 (17.2)	19,067 (17.7)	20,310 (18.9)	22,672 (21.1)
£18,000–£29,999	93,374 (21.7)	22,917 (21.3)	22,937 (21.3)	23,517 (21.9)	24,003 (22.3)
£30,000–£51,999	97,330 (22.6)	24,349 (22.6)	24,765 (23.0)	24,417 (22.7)	23,799 (22.1)
£52,000–£100,000	76,951 (17.9)	20,341 (18.9)	19,859 (18.5)	19,509 (18.1)	17,242 (16.0)
Greater than £100,000	20,653 (4.8)	5,782 (5.4)	5,547 (5.2)	4,896 (4.6)	4,428 (4.1)
Unknown	61,514 (14.3)	15,791 (14.7)	15,343 (14.3)	14,940 (13.9)	15,440 (14.4)
Townsend Deprivation Index (TDI)	−1.35 (3.07)	−1.29 (3.12)	−1.43 (3.02)	−1.40 (3.04)	−1.27 (3.10)
Education^†^					
Low	144,689 (33.6)	34,775 (32.3)	35,380 (32.9)	36,323 (33.8)	38,211 (35.5)
Medium	139,419 (32.4)	34,278 (31.8)	34,910 (32.5)	35,342 (32.8)	34,889 (32.4)
High	141,281 (32.8)	37,248 (34.6)	35,992 (33.5)	34,757 (32.3)	33,284 (30.9)
Unknown	4,958 (1.2)	1,355 (1.3)	1,236 (1.1)	1,167 (1.1)	1,200 (1.1)
**Health Behaviors**					
Smoking behavior					
Never	236,801 (55.0)	59,991 (55.7)	59,120 (55.0)	59,183 (55.0)	58,507 (54.4)
Previous	147,047 (34.2)	36,827 (34.2)	37,131 (34.5)	36,796 (34.2)	36,293 (33.7)
Current	44,421 (10.3)	10,286 (9.6)	10,778 (10.0)	11,105 (10.3)	12,252 (11.4)
Unknown	2,078 (0.5)	552 (0.5)	489 (0.5)	505 (0.5)	532 (0.5)
Alcohol consumption					
Never	18,227 (4.2)	5,361 (5.0)	4,438 (4.1)	4,305 (4.0)	4,123 (3.8)
Previous	14,296 (3.3)	3,514 (3.3)	3,427 (3.2)	3,496 (3.2)	3,859 (3.6)
Current	396,830 (92.2)	98,484 (91.5)	99,431 (92.5)	99,571 (92.5)	99,344 (92.3)
Unknown	994 (0.2)	297 (0.3)	222 (0.2)	217 (0.2)	258 (0.2)
Body Mass Index (BMI, kg/m^2^)					
Underweight (< 18.5)	1,955 (0.5)	402 (0.4)	438 (0.4)	493 (0.5)	622 (0.6)
Normal (18.5–24.9)	138,862 (32.3)	34,510 (32.1)	33,916 (31.5)	34,114 (31.7)	36,322 (33.8)
Overweight (25.0–29.9)	185,356 (43.1)	46,883 (43.5)	46,690 (43.4)	46,415 (43.1)	45,368 (42.2)
Obese (30+)	104,174 (24.2)	25,861 (24.0)	26,474 (24.6)	26,567 (24.7)	25,272 (23.5)
**Comorbidities**					
Cardiovascular diseases	21,467 (5.0)	4,256 (4.0)	4,767 (4.4)	5,476 (5.1)	6,968 (6.5)
Hypertension	111,279 (25.9)	24,989 (23.2)	26,538 (24.7)	28,313 (26.3)	31,439 (29.2)
Dyslipidemia	61,941 (14.4)	14,565 (13.5)	15,066 (14.0)	15,496 (14.4)	16,814 (15.6)
Diabetes	19,159 (4.5)	4,467 (4.1)	4,394 (4.1)	4,708 (4.4)	5,590 (5.2)
Cancer	38,351 (8.9)	9,199 (8.5)	9,137 (8.5)	9,241 (8.6)	10,774 (10.0)
HIV	415 (0.1)	201 (0.2)	91 (0.1)	67 (0.1)	56 (0.1)
Organ transplant	492 (0.1)	99 (0.1)	65 (0.1)	82 (0.1)	246 (0.2)
**Lab test**					
White blood cell count	6.85 (2.02)	6.36 (2.60)	6.62 (1.56)	6.91 (1.62)	7.53 (1.93)
Neutrophil count	4.20 (1.38)	3.18 (0.92)	3.88 (0.95)	4.39 (1.07)	5.33 (1.52)
Lymphocyte count	1.97 (1.13)	2.48 (1.99)	2.06 (0.50)	1.83 (0.45)	1.52 (0.42)
Neutrophil-to-lymphocyte ratio	2.32 (1.16)	1.34 (0.25)	1.89 (0.13)	2.40 (0.18)	3.65 (1.53)

Note: Q, quartile; SD, standard deviation; N, numbers; N/A, not available. ^*^The cut-off values for NLR quartiles were as follows: Q1 (< 1.65), Q2 (1.65–2.13), Q3 (2.13–2.74), and Q4 (> 2.74). ^†^Categorized as low: ‘None’, ‘Certificate of Secondary Education [CSEs]/Equivalent’, ‘National Vocational Qualification [NVQ]/Higher national Diploma [HND]/Higher National Cerfiticate [HNC]/Equivalent’, and ‘Other professional qualifications’; medium: ‘Ordinary [O] Levels/General Certificate of Secondary Education [GCSEs]/Equivalent’ and ‘Advanced/Advanced Subsidiary Levels [A/AS Levels] Equivalent’; and high: ‘College/University’.

## Association between NLR and any autoimmune diseases

**Fig. 1:**
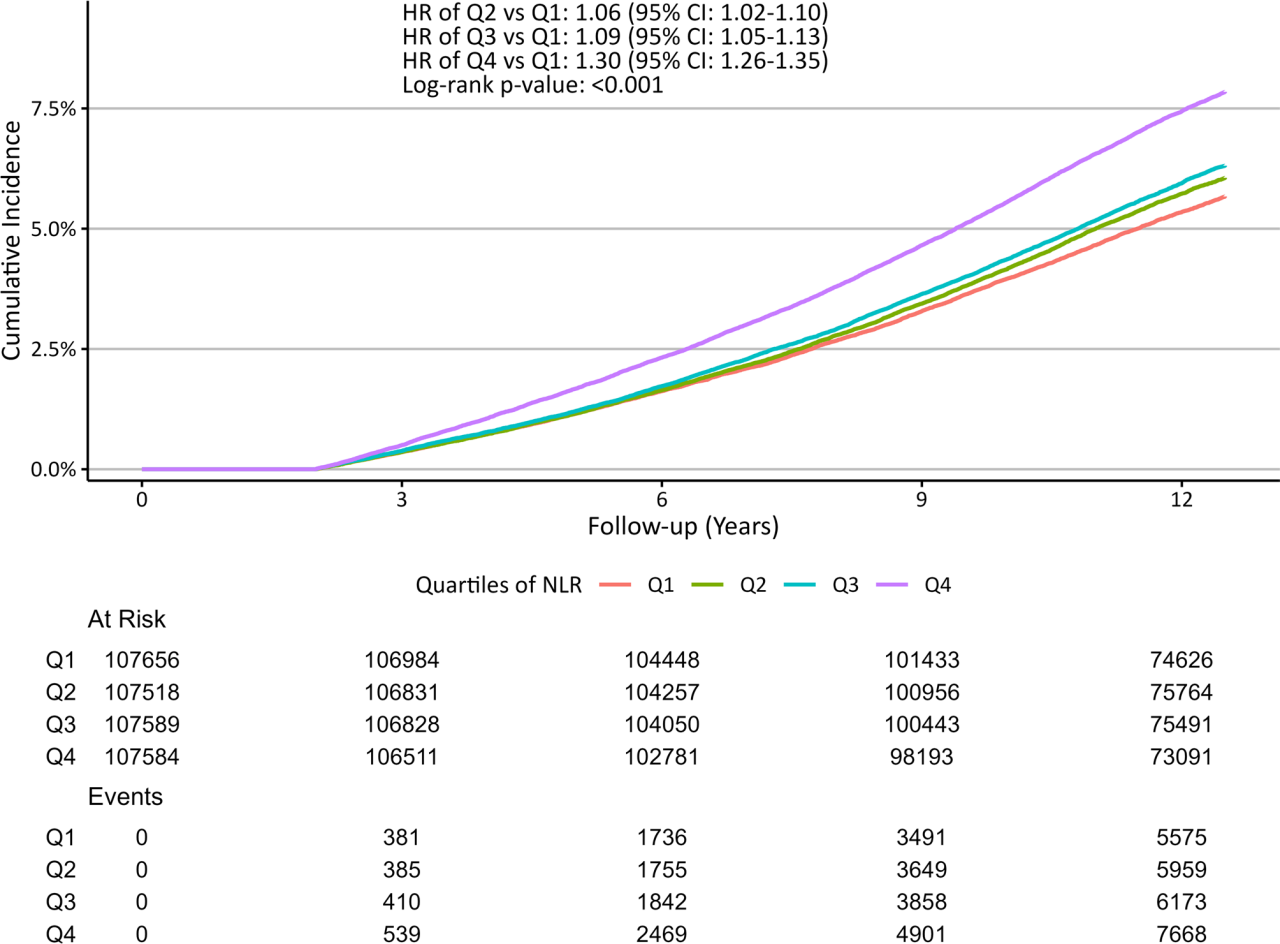
Cumulative incidence curve of any autoimmune disease by quartile increases of neutrophil-to-lymphocyte ratio. *Notes: The log-rank test and post-hoc pairwise comparisons were conducted across the four quartiles of NLR (six pairwise comparisons)*,* with Bonferroni correction*,* revealing significant differences across the quartiles.*

During follow-up, 27,571 events of autoimmune diseases were identified, with a median age at diagnosis of 69.2 years and a median time to diagnosis of 8.3 years (**Supplementary Table 2**). We identified that higher NLR was significantly associated with autoimmune disease risk (per quartile increase in NLR: HR 1.09, 95% CI 1.07–1.10, p–trend < 0.001) (Table 2). Compared to participants in Q1, the risk of any autoimmune disease increased across higher quartiles (HR_Q2 vs. Q1_ 1.06, 95% CI 1.02–1.10; HR_Q3 vs. Q1_ 1.09, 95% CI 1.05–1.13; HR_Q4 vs. Q1_ 1.30, 95% CI 1.26–1.35) (Table 2; Fig. 1). Participants in the highest NLR quartile exhibited significantly greater cumulative incidence compared to those in the lowest quartile (log-rank for overall *p* < 0.001, *p* < 0.001 for all post-hoc pairwise comparisons).

**Table 2:** Associations between neutrophil-to-lymphocyte ratio and any autoimmune diseases by subgroup and sensitivity analyses.

	Events	Q2 vs. Q1 aHR (95% CI)^*^	Q3 vs. Q1 aHR (95% CI)^*^	Q4 vs. Q1 aHR (95% CI)^*^	Per Q increase aHR (95% CI)^*^	*p*-trend	*p*-for-heterogeneity^†^
**Main analysis**	27,571	1.06 (1.02–1.10)	1.09 (1.05–1.13)	1.30 (1.26–1.35)	1.09 (1.08–1.10)	< 0.001	
**Subgroup analysis**							
Age							0.16
Age ≥ 65	8,724	0.99 (0.93–1.05)	1.05 (0.99–1.12)	1.31 (1.23–1.39)	1.09 (1.07–1.10)	< 0.001	
Age < 65	18,847	1.07 (1.03–1.12)	1.11 (1.06–1.15)	1.29 (1.24–1.34)	1.09 (1.08–1.11)	< 0.001	
Sex							0.71
Female	15,062	1.08 (1.03–1.14)	1.09 (1.04–1.14)	1.28 (1.22–1.34)	1.08 (1.07–1.10)	< 0.001	
Male	12,509	1.04 (0.98–1.09)	1.09 (1.03–1.15)	1.32 (1.25–1.39)	1.09 (1.08–1.11)	< 0.001	
**Sensitivity analysis**							
5-year lag time	22,027	1.07 (1.03–1.12)	1.10 (1.06–1.15)	1.29 (1.24–1.34)	1.09 (1.07–1.10)	< 0.001	
Excluding patients with IC	24,389	1.06 (1.02–1.10)	1.10 (1.06–1.14)	1.32 (1.27–1.37)	1.09 (1.08–1.11)	< 0.001	

Note: Q, quartile; HR, hazard ratio; CI, confidence interval; IC, immunocompromised. *Adjusted for sociodemographic variables (age, sex, race/ethnicity, income, education, and TDI), health behaviors (smoking, alcohol consumption, and BMI), comorbidities (cardiovascular diseases, hypertension, dyslipidemia, diabetes, and cancer), and white blood cell counts. ^**†**^Calculated from the per-quartile estimate between subgroups.

## Association between NLR and individual autoimmune disease

**Fig. 2:**
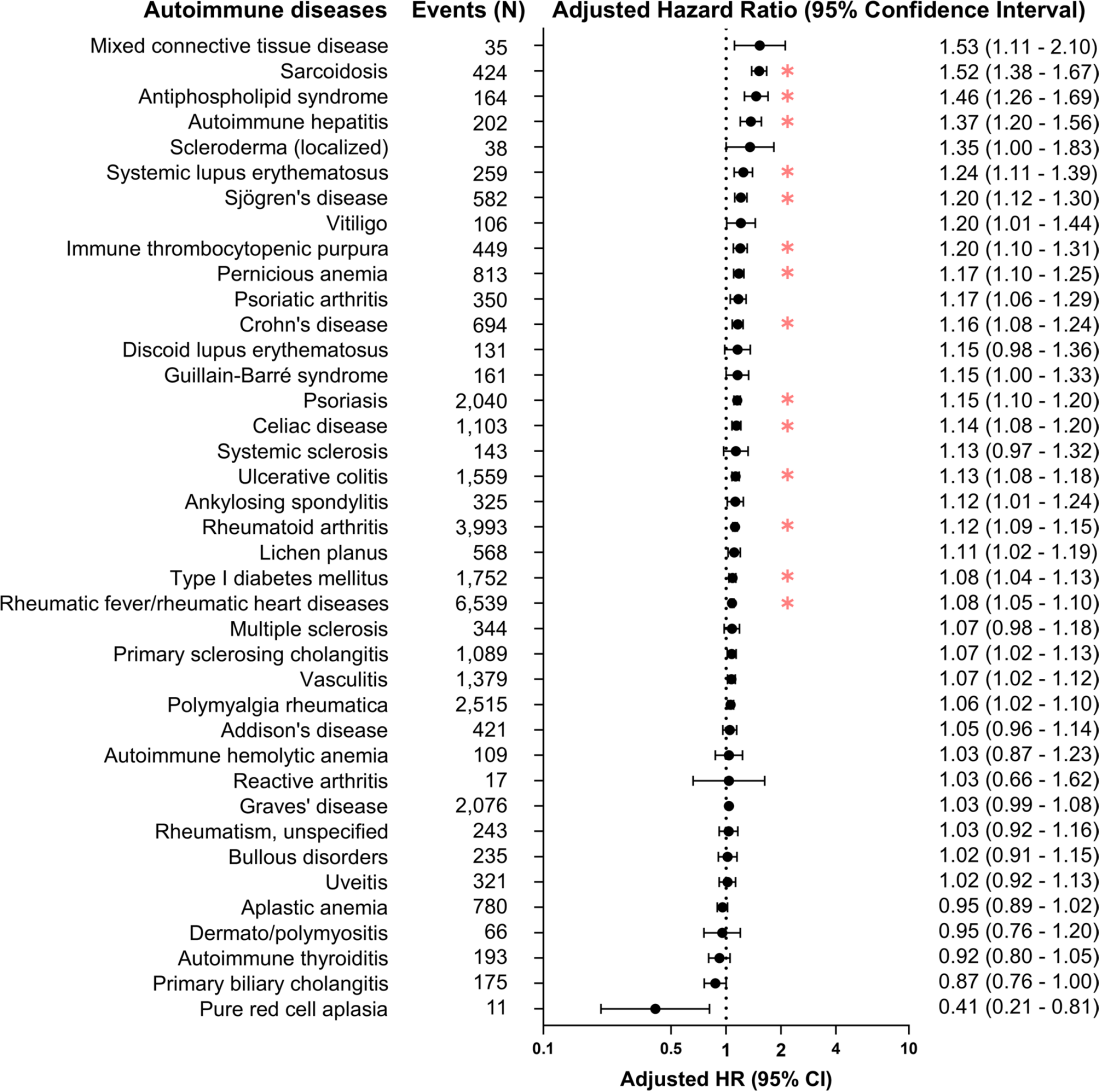
Associations between neutrophil-to-lymphocyte ratio and individual autoimmune diseases. Notes: Adjusted hazard ratios and their 95% confidence intervals were estimated by multivariable Cox proportional hazard model for per quartile neutrophil-to-lymphocyte increase. Asterisk (*) indicates statistical significance after Bonferroni correction.

Among 39 individual autoimmune diseases assessed, we found that elevated NLR was associated with 14 autoimmune diseases: sarcoidosis (HR per NLR quartile increase 1.52, 95% CI 1.38–1.67), antiphospholipid syndrome (1.46, 95% CI 1.26–1.69), autoimmune hepatitis (1.37, 95% CI 1.20–1.56), systemic lupus erythematosus (1.24, 95% CI 1.11–1.39), Sjögren’s disease (1.20, 95% CI 1.12–1.30), immune thrombocytopenic purpura (1.20, 95% CI 1.10–1.31), pernicious anemia (1.17, 95% CI 1.10–1.25), Crohn’s disease (1.16, 95% CI 1.08–1.24), psoriasis (1.15, 95% CI 1.10–1.20), celiac disease (1.14, 95% CI 1.08–1.20), ulcerative colitis (1.13, 95% CI 1.08–1.18), rheumatoid arthritis (1.12, 95% CI 1.09–1.15), type 1 diabetes mellitus (1.08, 95% CI 1.04–1.13), and rheumatic fever/rheumatic heart diseases (1.08, 95% CI 1.05–1.10) (Fig. 2).

## Non-linear association and threshold assessment of NLR in autoimmune disease risk

**Fig. 3:**
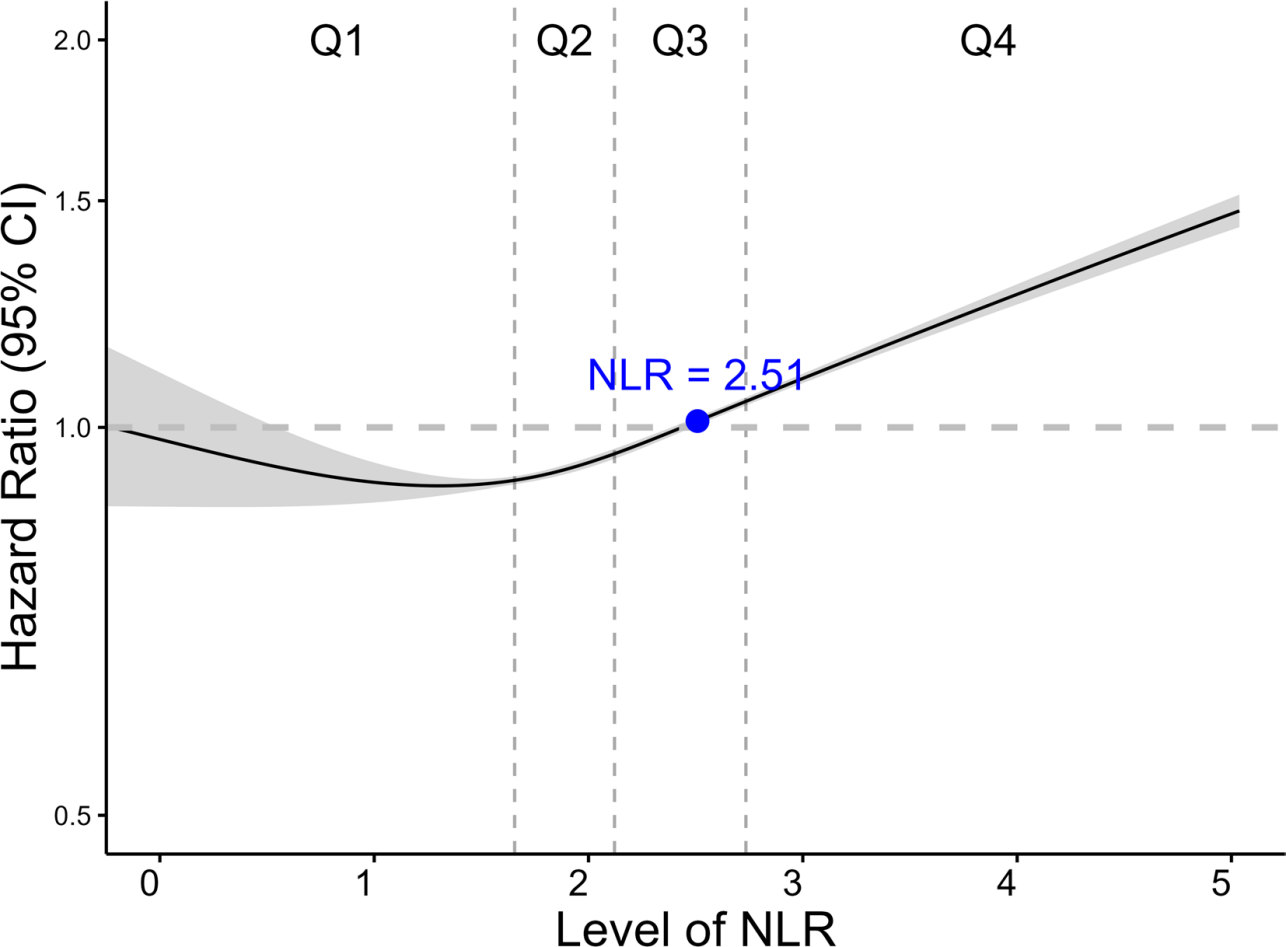
Spline curve of neutrophil-to-lymphocyte ratio and risk of autoimmune diseases. *Notes: The NLR value of 2.51 with blue dot represents the point where the lower confidence interval of the hazard ratio exceeds 1.*

A spline curve depicting the association between NLR and autoimmune disease risk demonstrated a non–linear association (p-for-non-linearity < 0.001) (Fig. 3). The risk of autoimmune diseases increased markedly at NLR values exceeding 2.51. Age-stratified splines yielded nearly identical shapes (**Supplementary Fig. 2**). The NLR cut-off value was 2.50 in participants under 65 years and 2.68 in those aged 65 or older, showing a marginal right-shift in older adults.

## Subgroup and sensitivity analyses

In the age-based subgroup analysis of any autoimmune diseases (Table 2), no significant interaction was observed between participants under 65 years and those aged 65 or older (HR_< 65y_ 1.09, 95% CI 1.08–1.11; HR_≥65y_ 1.09, 95% CI 1.07–1.10; p-for-interaction = 0.16). Similarly. the sex-stratified analysis indicated no significant heterogeneity between female and male (HR_female_ 1.08, 95% CI 1.07–1.10; HR_male_ 1.09, 95% CI 1.08–1.11; p-for-interaction = 0.71).

In the age-stratified analyses of individual autoimmune diseases, associations between NLR and autoimmune diseases were mostly similar in both age groups (**Supplementary Table 3**). The associations were stronger in individuals under 65 years for sarcoidosis (HR_≥65y_ 1.18, 95% CI 0.98–1.43; HR_< 65y_ 1.62, 95% CI 1.46–1.80; p-for-interaction < 0.01) and autoimmune hepatitis (HR_≥65y_ 1.10, 95% CI 0.86–1.40; HR_< 65y_ 1.49, 95% CI 1.27–1.74; p-for-interaction = 0.04). Conversely, the NLR associations were stronger in ≥ 65 years for immune thrombocytopenic purpura (HR_≥65y_ 1.38, 95% CI 1.17–1.62; HR_< 65y_ 1.13, 95% CI 1.02–1.25; p-for-interaction = 0.04).

In the sex-stratified analyses, significant heterogeneity was observed for ankylosing spondylitis, with a stronger association in males (HR_male_ 1.24, 95% CI 1.08–1.43; HR_female_ 1.00, 95% CI 0.86–1.16; p-for-interaction = 0.03).

For sensitivity analyses, extending the lag period to 5 years yielded consistent results (HR_lag-5y_ 1.09, 95% CI 1.07–1.10) (Table 2). The associations between NLR and individual autoimmune disease remained statistically significant after varying a lag to 5-year, but associations of systemic lupus erythematosus (HR_lag-5y_ 1.18, 95% CI 1.04–1.34) and Sjögren’s disease (HR_lag-5y_ 1.13, 95% CI 1.03–1.23) were not significant after Bonferroni correction (**Supplementary Table 5**). Similarly, excluding immunocompromised patients (*N* = 39,010) did not alter the association between NLR and autoimmune diseases (HR_excluding immunocompromised_ 1.09, 95% CI 1.08–1.11) (Table 2). For individual analysis of excluding immunocompromised patients yielded results consistent with the primary analysis, with less than 10% variation in HR for most conditions, except for pure red cell aplasia, which exhibited greater variability due to the small number of events.

Analyses across NLR quartiles demonstrated consistent associations between NLR and individual autoimmune diseases (**Supplementary Table 6**). While no significant association was observed when comparing Q2 to Q1 in the individual analysis, the associations became significant with higher quartiles, suggesting that the elevated NLR is strongly associated with an increased risk of autoimmune diseases.

## Discussion

In this large population-based cohort of over 430,000 individuals, this study is, to our knowledge, the first to demonstrate a positive association between an elevated NLR and the increased risk of incident autoimmune diseases using a prospective design. Participants in the highest NLR quartile exhibited a 30% greater risk of developing autoimmune diseases, compared to those in the lowest quartile. Notably, a non-linear association was observed between NLR and any autoimmune disease, with risk increasing significantly at NLR values above 2.51. We identified 14 specific autoimmune diseases associated with elevated NLR. Subgroup analyses revealed heterogeneity across age and sex, and our findings remained consistent in sensitivity analyses. Although the associations for SLE and Sjögren’s disease were attenuated in the 5-year lag analysis, this may be partly explained by reduced statistical power due to a smaller number of incident cases after excluding early events.

Our findings support a positive dose-response relationship between NLR and incident autoimmune disease, owing to a pro-inflammatory environment that may promote autoimmune disease development. Our NLR quartiles align closely with clinically relevant thresholds: Q1 (< 1.65) lies within the normal range; Q2 (1.65–2.13) represents the upper end of normal; Q3 (2.13–2.74) corresponds to a subclinical “grey zone” of mild inflammation; and Q4 (> 2.74) exceeds the typical normal range, indicating a distinctly elevated inflammatory state^6^. Consistent with these categories, our analyses showed a significant dose-response association across quartiles (p-trend < 0.001), with progressively higher autoimmune disease risk observed from Q2 through Q4 compared to Q1. Additionally, our spline analysis identified a risk threshold at NLR 2.51, lied in the upper part of Q3, indicating the transition from subclinical inflammation into a clear pathological inflammatory state (Q4). These findings reinforce that NLR could serve as a practical clinical marker to identify individuals who may benefit from closer monitoring or earlier intervention to prevent autoimmune disease progression.

Previous studies proposed that NLR could be a potential diagnostic or prognostic marker of diagnosis, as studies comparing NLR levels in patients with autoimmune disease and healthy controls found significantly higher NLR levels in the patients^15–20^. While these earlier studies offered cross-sectional or retrospective insights with limited sample sizes, our research further establishes a prospective link between NLR and the development of autoimmune diseases. These findings raise the possibility that NLR could serve as an early indicator of immune dysregulation, although additional validation is needed to assess its utility and predictive value in clinical practice.

Excessive neutrophils can promote autoimmunity by producing reactive oxygen species (ROS), secreting pro-inflammatory cytokines and chemokines, and forming neutrophil extracellular traps (NETs). Dysregulated NET formation exposes intracellular components like DNA and proteins, potentially triggering an autoimmune response^21^. In this study, we identified several autoimmune diseases associated with elevated NLR, and these can be supported by the role of NETs^22^. In antiphospholipid syndrome, NET-derived oxidized phospholipids and β2-glycoprotein I complexes stimulate pathogenic antiphospholipid antibodies^23^, promoting thrombosis and endothelial injury^24^. In autoimmune hepatitis, neutrophil-derived ROS and proteases exacerbate hepatocellular injury, linking hepatic inflammation to systemic autoimmunity^25^. Similarly, systemic lupus erythematosus involves neutrophilic hyperactivity via NETosis, which exposes autoantigens like double-stranded DNA^26^. Rheumatoid arthritis is also associated with NETs formation^27^. NET-associated citrullinated peptides trigger autoantibody production and contribute to synovial inflammation and joint tissue damage.

The Th17/IL-17 axis plays a pivotal role in promoting neutrophil recruitment and activation, resulting in inflammation across various tissues. The heightened Th17/IL-17 signaling may help explain the observed elevations in NLR. For instance, sarcoidosis is characterized by granuloma formation linked to Th17 cells, and overproduction of neutrophils can exacerbate organ damage^28^. In psoriasis, IL-17 promotes keratinocyte hyperproliferation and neutrophil infiltration into skin lesions, intensifying the inflammatory response^29^. Similarly, in Crohn’s disease and ulcerative colitis, an amplified Th17 response promotes neutrophil-mediated mucosal injury in the gastrointestinal tract^29^. In Sjögren disease, Th17 and IL-17 have been identified at both the protein and mRNA levels in inflamed salivary glands and peripheral blood, suggesting an active Th17-driven inflammatory process^30^. This elevated Th17 activity correlates with a decreased number of T regulatory cells and higher concentrations of other interleukins (IL-21, IL-22, IL-23)^31^. In type 1 diabetes, upregulation of Th17 immunity has been observed in both peripheral blood and lymph nodes, reflecting a pronounced inflammatory profile^32^. Moreover, dysregulation of the interaction between Th17 and T regulatory cells further disrupts immune homeostasis, potentially accelerating the autoimmune destruction of pancreatic β-cells^33^. Collectively, these processes shift the immune balance toward a more neutrophil-dominant state, manifesting as an elevated NLR. Although our findings are consistent with activation of the Th17/IL-17 axis, this inference should be interpreted cautiously because our study did not include direct measurements of related cytokines or NET formation. Accordingly, we view NLR as a proxy for broader systemic inflammatory dysregulation rather than as evidence of a single causal pathway.

We found that sarcoidosis and autoimmune hepatitis, which frequently manifest in younger or middle-aged adults, showed a higher risk in individuals under 65 compared to those over 65. These findings align with the known disease epidemiology and may indicate that an elevated NLR could be associated with the dysregulation of Th17-mediated immune responses, potentially influencing disease development^34,35^. Conversely, in older adults, several factors, including multimorbidity, polypharmacy, and immunosenescence can alter the inflammatory process, affecting the increased occurrence of immune thrombocytopenic purpura in this subgroup^36^. Although NLR increases with aging^37^, we observed that most autoimmune diseases did not demonstrate significant age interactions. This lack of age interactions suggests that immunologic mechanisms beyond age-related inflammation may affect these associations, warranting further investigation.

In sex-stratified analysis of ankylosing spondylitis, the Th17/IL-17 pathway and excessive NET formation are both implicated in ankylosing spondylitis pathogenesis,^29^ and sex-specific differences in these immune processes may help explain the stronger association in males. For instance, sex hormones can differentially modulate Th17 cell expansion and IL-17 production, as well as influence the regulation of NET release^38^. These heightened pathways in men may lead to higher inflammatory activity, thereby amplifying the association between elevated NLR and ankylosing spondylitis.

## Strengths and limitations

This study possesses several strengths. First, the prospective nature of this study enables to establish a temporal association between elevated NLR and the subsequent risk of autoimmune diseases. Second, we assessed a broad range of 39 autoimmune diseases, providing comprehensive evidence with sufficient statistical power, including rare diseases that may be overlooked in smaller cohorts. Third, a range of sensitivity analyses, including an extended lag period of up to five years and the exclusion of immunocompromised individuals, supports our main findings.

However, limitations warrant attention. First, we relied on a single baseline measurement of NLR, potentially overlooking fluctuations in inflammatory status over time. Single-timepoint NLR measurements may therefore misclassify participants who have dynamic inflammatory profiles. Nevertheless, NLR is considered a relatively stable marker, less affected by acute conditions compared to total WBC or counts of WBC subsets^39^. Future studies incorporating serial NLR assessments are needed to more accurately capture cumulative inflammatory exposure over time. One other limitation of using CBC data is that it quantifies total neutrophils and lymphocytes but does not provide specificity for different B- or T-cell subpopulations. This lack of detail may obscure important immunological variations contributing to NLR, potentially limiting its ability to fully capture immune dysregulation in autoimmune diseases. Second, although we adjusted for multiple confounders, the potential for residual or unmeasured confounding cannot be ruled out. Third, autoimmune disease diagnoses were primarily identified through ICD codes, which may underestimate events diagnosed exclusively in primary care settings and introduce potential misclassification. However, given that autoimmune diseases often require specialist evaluation, this limitation is likely mitigated. For example, a Swedish validation study reported positive predictive values for psoriasis and psoriatic arthritis ranging from 81 to 100% and 63–92%, respectively, showing reasonable validity^40^. Finally, the identified NLR threshold may not be universally applicable, given that the study was conducted among predominantly middle-aged and older White participants residing in the UK, potentially limiting its relevance to other populations. Future investigations should include more diverse cohorts, particularly younger age groups where many autoimmune diseases first manifest. This should involve repeated NLR measurements and rigorous validation of autoimmune disease diagnoses to strengthen these findings.

## Conclusion

In this large prospective population-based cohort, this study is, to our knowledge, the first to demonstrate a significant positive association between NLR and a comprehensive list of autoimmune diseases, particularly for sarcoidosis, antiphospholipid syndrome, and autoimmune hepatitis. Given its low cost and routine availability as part of CBC testing, NLR represents a pragmatic and readily implementable clinical tool for early risk stratification in primary care settings. Future studies are warranted to explore the additive clinical value of NLR alongside established biomarkers such as C-reactive protein and autoantibody panels, thereby refining its role in the early diagnosis and management of autoimmune diseases.

### Methods

#### Data sources and study design

The UK Biobank is a large-scale biomedical database of approximately 500,000 adults aged 40–69 years, recruited between 2006 and 2010 (https://www.ukbiobank.ac.uk/)^41^. Data collection included physical examinations, biological samples, and self-reported information from touchscreen questionnaires and computer-assisted interviews, covering demographics, lifestyle, and medical history. Health outcomes were obtained from self-reports and linked electronic health records, including hospital inpatient records, primary care records, and death and cancer registries.

Among 502,389 study participants who consented to data use, we excluded individuals had missing body mass index (BMI) or Townsend Deprivation Index (TDI) data (*n* = 10,741), had any pre-existing diagnosis of autoimmune diseases at baseline (*n* = 34,745), or those without a record of complete blood cell (CBC) count results (*n* = 21,827). We applied a two-year lag period to minimize reverse causality by excluding participants with follow-up less than two years (*n* = 4,729). The final study population comprised 430,347 participants (**Supplementary Fig. 1**). Hematology data were assessed by Beckman Coulter LH750 at the UK Biobank central laboratory within 24 h of blood collection^42^. The NLR was calculated by dividing neutrophil counts by lymphocyte counts, was categorized into quartiles based on the distribution of NLR in the final cohort: Q1 (NLR < 1.65), Q2 (1.65–2.13), Q3 (2.13–2.74), and Q4 (> 2.74). The project was ethically approved by the North West Multi-Centre Research Ethics Committee and the Institutional Review Board of the National Institute on Aging, Intramural Research Program. It was also endorsed by the UK Biobank access management team (application #77963). All methods were performed in accordance with relevant guidelines and regulations.

### Outcome ascertainment

Autoimmune diseases for this study were selected based on the prior literature and the Autoimmune Registry^43^. We identified 39 autoimmune diseases using ICD-9 and − 10 codes from hospital inpatient records: Addison’s disease, ankylosing spondylitis, antiphospholipid syndrome, aplastic anemia, autoimmune hemolytic anemia, autoimmune hepatitis, autoimmune thyroiditis, bullous disorders, Celiac’s disease, Crohn’s disease, dermato/polymyositis, discoid lupus erythematosus, Graves’ disease, Guillain-Barré syndrome, immune thrombocytopenic purpura, lichen planus, mixed connective tissue disease, multiple sclerosis, pernicious anemia, polymyalgia rheumatica, primary biliary cholangitis, primary sclerosing cholangitis, psoriatic arthritis, psoriasis, pure red cell aplasia, reactive arthritis, rheumatoid arthritis, rheumatic fever/rheumatic heart diseases, rheumatism, sarcoidosis, scleroderma, Sjögren’s disease, systemic lupus erythematosus, systemic sclerosis, type 1 diabetes mellitus, ulcerative colitis, uveitis, vasculitis, and vitiligo (**Supplementary Table 1)**. We defined the primary outcome as the occurrence of any autoimmune diseases, while the secondary outcome assessed the occurrence of an individual autoimmune disease. Participants were followed up from the date of recruitment until the earliest occurrence of the following events: the date of outcome occurrence, death, loss to follow-up, or the end of the study period in October 2021.

### Potential confounders

We evaluated a range of potential confounders, including socio-demographic, lifestyle-related, and clinical factors. Socio-demographic variables included baseline age, sex, race/ethnicity, income level, education level, and TDI^44^. Lifestyle-related factors included smoking status, alcohol consumption, and BMI. Clinical factors encompassed baseline comorbidities, including cardiovascular diseases (a composite of atrial fibrillation, heart failure, myocardial infarction, stroke, transient ischemic attack), hypertension, diabetes, dyslipidemia, and cancer (**Supplementary Table 1**).

### Statistical analysis

We summarized descriptive statistics as means and standard deviations (SD) for continuous variables and as frequencies and proportions for categorical variables. For the primary outcome, we estimated adjusted hazard ratios (HRs) with corresponding 95% confidence intervals (CI) using Cox proportional hazard regression model, adjusting for socio-demographic characteristics, lifestyle-related factors, clinical confounders, and the total WBC count to account for any potential influence of overall leukocyte levels. We presented a cumulative incidence plot with log-rank test and post-hoc pairwise comparisons adjusted by Bonferroni correction to investigate differences between survival curves across NLR quartiles (0.05/6 = 0.0083). Additionally, we presented the association between NLR level and the risk of autoimmune diseases using Cox regression model with natural cubic splines (degree-of-freedom = 3) to account for potential non-linear associations. We defined the threshold for a statistically significant increase in autoimmune disease risk as the NLR value at which the lower bound of the 95% confidence interval for the HR first exceeded 1. For the secondary outcome, Cox regression models were applied individually for each of the 39 autoimmune diseases with Bonferroni correction (0.05/39 = 0.0013). All analyses were performed using R software (version 4.3.3).

### Subgroup and sensitivity analyses

We conducted subgroup analyses to explore potential heterogeneity by age (≥ 65 vs. <65 years) and sex (female vs. male). The p-value for interaction was calculated to assess the statistical significance of the interaction between subgroups and NLR. Interaction effects were considered statistically significant if the p-for-interaction was below 0.05.

Sensitivity analyses were performed to test robustness: (1) extending predefined lag period from two to five years, (2) excluding immunocompromised individuals (participants with prior cancer, human immunodeficiency virus, or organ transplantation, identified through ICD-9/10 codes), who may have a higher predisposition to developing autoimmune diseases.

## Supplementary Information

Below is the link to the electronic supplementary material.

Supplementary Material 1

## Data Availability

Interested researchers are welcome to contact the corresponding author (Dongwon Yoon) to discuss data access options.

## Declarations

### Patient and public involvement

As we used de-identified data in our analyses, the need to obtain informed consent was waived. No patients were involved in establishing the research question or outcome measures or in developing plans for recruitment, design, or implementation of the study. No patients were asked for advice on interpretation or documentation of results. There are no plans to disseminate the research results to study participants or the relevant patient community.

### Transparency statement

The lead authors affirm that this manuscript is an honest, accurate, and transparent account of the study being reported; that no important aspects of the study have been omitted; and that any discrepancies from the study as planned (and, if relevant, registered) have been explained.

### Dissemination to participants and related patient and public communities

We have no plans to disseminate the results of the research to study participants or the relevant patient community. As anonymized data were used in this study, the authors had no direct contact information of the study participants to disseminate the results of the research.
