# Supplementary material for: Elevated neutrophil-to-lymphocyte ratio and the incidence of autoimmune diseases: evidence from a large prospective cohort study
Source: Sci Rep. 2026 Jan 6;16:667. doi: 10.1038/s41598-025-21188-y (PMC12780031; doi:10.1038/s41598-025-21188-y)
Supplement: Supplementary file 1 — Supplementary Material 1 [file 41598_2025_21188_MOESM1_ESM.docx]

**Supplementary Materials**

**Elevated Neutrophil-to-lymphocyte Ratio and the Incidence of
Autoimmune Diseases**

Dongwon Yoon, Choa Yun, Isabel Beerman, May A. Beydoun, Lenore J. Launer, Minkyo Song

[**Supplementary Table 1**. Codes used to define autoimmune diseases, and comorbidities 2](#_Toc204987751)

[**Supplementary Table 2.** Descriptive statistics of incident autoimmune diseases during follow-up 4](#_Toc204987752)

[**Supplementary Table 3.** Association between neutrophil-to-lymphocyte ratio and autoimmune diseases by age group (≥65 vs. <65 years) 5](#_Toc204987753)

[**Supplementary Table 4.** Association between neutrophil-to-lymphocyte ratio and autoimmune diseases by sex 7](#_Toc204987754)

[**Supplementary Table 5.** Sensitivity analyses results on the association between neutrophil-to-lymphocyte ratio and autoimmune diseases 9](#_Toc204987755)

[**Supplementary Table 6.** Association between neutrophil-to-lymphocyte ratio and autoimmune diseases across quartiles 11](#_Toc204987756)

[**Supplementary Figure 1.** Flow chart of population selection criteria in the UK biobank database 13](#_Toc204987761)

[**Supplementary Figure 2.** Spline curve of neutrophil-to-lymphocyte ratio and risk of autoimmune diseases by age group 14](#_Toc204987762)

# **Supplementary Table 1**. Codes used to define autoimmune diseases, and comorbidities

| **Categories** | **Codes** | | |
| --- | --- | --- | --- |
| **Autoimmune disease*** | **ICD-9 codes** | **ICD-10 codes** | **Self-report** |
| Addison's disease | 255.4 | E27.1, E27.2, E27.4 | 1234 |
| Ankylosing spondylitis | 720.0 | M45 | 1313 |
| Antiphospholipid syndrome |  | D68.6 | 1564 |
| Aplastic anemia | 284.9 | D61.9 | 1332 |
| Autoimmune hemolytic anemia | 283.0 | D59.1 |  |
| Autoimmune hepatitis | 571.42 | K75.4 |  |
| Autoimmune thyroiditis (Hashimoto’s thyroiditis) | 245.2 | E06.3 |  |
| Bullous disorders |  | L10-L14 |  |
| Celiac disease | 579.0 | K90.0 | 1456 |
| Crohn's disease | 555 | K50 | 1462 |
| Ulcerative colitis | 556 | K51 | 1463 |
| Dermato/polymyositis | 710.3, 710.4 | M33 | 1383, 1480, 1481 |
| Discoid lupus erythematosus | 695.4 | L93.0, L93.2 |  |
| Diabetes mellitus (Type I) | 250.01 | E10 | 1222 |
| Graves' disease | 242.0 | E05.0, E05.8, E05.9 | 1522 |
| Guillain-Barré syndrome | 357.0 | G61.0 | 1256 |
| Immune thrombocytopenic purpura | 287.31 | D69.3 |  |
| Lichen planus | 697.0 | L43 | 1549 |
| Mixed connective tissue disease | 710.8 | M35.1 |  |
| Multiple sclerosis | 340 | G35 | 1261 |
| Pernicious anemia | 281.0 | D51.0 |  |
| Polymyalgia rheumatica | 725 | M35.3 | 1377 |
| Primary biliary cholangitis | 571.6 | K74.3 | 1506 |
| Primary sclerosing cholangitis | 576.1 | K83.0 | 1475 |
| Psoriasis | 696.0, 696.1 | L40 | 1453 |
| Psoriatic arthritis | 696.0 | M07 | 1477 |
| Pure red cell aplasia | 284.8 | D60 |  |
| Reactive arthritis (Reiter’s disease) | 99.3 | M02.3 |  |
| Rheumatic fever / rheumatic heart diseases | 390, 391, 392 | I00-I02, I05-I09 | 1479 |
| Rheumatism, unspecified |  | M79.0 |  |
| Rheumatoid arthritis | 714.0, 714.1, 714.2, 714.3,714.4, 714.8, 714.9 | M05, M06, M08 | 1464 |
| Sarcoidosis | 135 | D86 | 1371 |
| Scleroderma (localized) | 701.0 | L94.0 | 1384 |
| Sjögren’s disease | 710.2 | M35.0 | 1382 |
| Systemic lupus erythematosus | 710.0 | L93, M32 | 1381 |
| Systemic sclerosis | 710.1 | M34 | 1384 |
| Uveitis | 364.3 | H20.9 |  |
| Vasculitis | 136.1, 287.0, 446.0, 446.1, 446.2, 446.4, 446.5, 446.7, 447.5 | N00.3-N00.5, N00.7, N01.2-N01.5, N01.7, N02.2-N02.7, N03.2-N03.5, N03.7, N04.2-N04.5, N04.7, N05.2-N05.5, N05.7, N06.2-N06.5, N06.7, N07.2-N07.5, N07.7, I73.1, M31.0, M31.3, M31.4, M31.5, M31.6, M31.7, M31.9, I67.7, I68.1, I68.2, I77.6, M30.0, M30.1, M30.2, M30.3 M30.8, L95, L95.0, L95.8, L95.9, M05.2, M35.2, D69.0 | 1372, 1376, 1378, 1379, 1380 |
| Vitiligo | 709.01, 374.53 | L80, H02.739 | 1661 |
| **Comorbid medical conditions^*^** | **Definition** | | |
| Cardiovascular disease | The presence of the first date of occurrence of cardiovascular diseases, as recorded in the UK Biobank using mapped ICD-10 codes (I21, I22, I48, I50, I60-I64, G45) before baseline. These records were collected through either self-report at any assessment center, inpatient hospital data, primary care, or death record data. | | |
| Hypertension | The presence of the first date of occurrence of hypertension, as recorded in the UK Biobank using mapped ICD-10 codes (I10-I15) before baseline. These records were collected through either self-report at any assessment center, inpatient hospital data, primary care, or death record data. | | |
| Dyslipidemia | The presence of the first date of occurrence of dyslipidemia, as recorded in the UK Biobank using mapped ICD-10 codes (E78) before baseline. These records were collected through either self-report at any assessment center, inpatient hospital data, primary care, or death record data. | | |
| Diabetes | The presence of the first date of occurrence of diabetes, as recorded in the UK Biobank using mapped ICD-10 codes (E11-E14) before baseline. These records were collected through either self-report at any assessment center, inpatient hospital data, primary care, or death record data. | | |
| Cancer | Having a diagnosis of cancer before baseline from the national cancer registry | | |
| Immunocompromised | Having a diagnosis of cancer or the presence of the first date of occurrence of human immunodeficiency virus or organ transplant, as recorded in the UK Biobank using mapped ICD-10 codes (B20-B24 or Z94, T86, V42) before baseline. These records were collected through either self-report at any assessment center, inpatient hospital data, primary care, or death record data. | | |

**Abbreviation:** ICD: International Classification of Diseases

^*^Comorbidities were identified using the First Occurrence Health Outcomes data set of each disease, based on the mapped ICD-10 codes within the UK Biobank. This data encompasses self-reports from assessment centers, hospitalization records, primary care, and death records.

# **Supplementary Table 2.** Descriptive statistics of incident autoimmune diseases during follow-up

| **Autoimmune diseases** | **Number of events** | **Median age-at-diagnosis (overall)** | **Median time-to-diagnosis (overall)** |
| --- | --- | --- | --- |
| Any autoimmune diseases | 27,571 | 69.2 | 8.3 |
| Addison's disease | 421 | 66.7 | 9.1 |
| Ankylosing spondylitis | 325 | 68.9 | 8.3 |
| Antiphospholipid syndrome | 164 | 65.1 | 8.7 |
| Aplastic anemia | 780 | 70.6 | 8.5 |
| Autoimmune hemolytic anemia | 109 | 69.0 | 8.1 |
| Autoimmune hepatitis | 202 | 68.1 | 8.1 |
| Autoimmune thyroiditis | 193 | 63.7 | 8.4 |
| Bullous disorders | 235 | 70.7 | 8.6 |
| Celiac disease | 1,103 | 65.9 | 7.6 |
| Crohn's disease | 694 | 66.1 | 7.9 |
| Dermato/polymyositis | 66 | 68.8 | 7.6 |
| Discoid lupus erythematosus | 131 | 67.2 | 7.4 |
| Graves' disease | 2,076 | 67.9 | 8.1 |
| Guillain-Barré syndrome | 161 | 66.7 | 7.1 |
| Immune thrombocytopenic purpura | 449 | 68.6 | 7.9 |
| Lichen planus | 568 | 68.0 | 8.5 |
| Mixed connective tissue disease | 35 | 65.2 | 5.3 |
| Multiple sclerosis | 344 | 62.4 | 7.4 |
| Pernicious anemia | 813 | 68.6 | 7.6 |
| Polymyalgia rheumatica | 2,515 | 72.6 | 9.1 |
| Primary biliary cholangitis | 175 | 67.0 | 7.4 |
| Primary sclerosing cholangitis | 1,089 | 70.7 | 9.0 |
| Psoriasis | 2,040 | 68.0 | 8.9 |
| Psoriatic arthritis | 350 | 65.0 | 8.6 |
| Pure red cell aplasia | 11 | 68.6 | 4.6 |
| Reactive arthritis | 17 | 65.3 | 9.5 |
| Rheumatic fever/rheumatic heart diseases | 6,539 | 72.1 | 9.5 |
| Rheumatism, unspecified | 243 | 60.4 | 3.1 |
| Rheumatoid arthritis | 3,993 | 69.0 | 8.3 |
| Sarcoidosis | 424 | 65.2 | 8.3 |
| Scleroderma (localized) | 38 | 66.6 | 5.9 |
| Sjögren’s disease | 582 | 67.9 | 8.0 |
| Systemic lupus erythematosus | 259 | 67.0 | 8.0 |
| Systemic sclerosis | 143 | 67.6 | 7.5 |
| Type I diabetes mellitus | 1,752 | 67.0 | 6.8 |
| Ulcerative colitis | 1,559 | 66.4 | 7.6 |
| Uveitis | 321 | 68.7 | 8.8 |
| Vasculitis | 1,379 | 70.1 | 8.4 |
| Vitiligo | 106 | 67.0 | 9.2 |

# **Supplementary Table 3.** Association between neutrophil-to-lymphocyte ratio and autoimmune diseases by age group (≥65 vs. <65 years)

| **Autoimmune diseases** | **Age ≥ 65** | | **Age < 65** | | **p-for-interaction** |
| --- | --- | --- | --- | --- | --- |
|  | **No of events** | **HR (95% CI)** | **No of events** | **HR (95% CI)** |  |
| Mixed connective tissue disease | 6 | 1.22 (0.58–2.58) | 29 | 1.61 (1.13–2.29) | 0.64 |
| Sarcoidosis | 85 | 1.18 (0.98–1.43) | 339 | 1.62 (1.46–1.80)* | <.01 |
| Antiphospholipid syndrome | 29 | 1.60 (1.11–2.30) | 135 | 1.42 (1.21–1.68)* | 0.84 |
| Autoimmune hepatitis | 54 | 1.10 (0.86–1.40) | 148 | 1.49 (1.27–1.74)* | 0.04 |
| Scleroderma (localized) | 9 | 1.24 (0.67–2.28) | 29 | 1.39 (0.98–1.96) | 0.85 |
| Systemic lupus erythematosus | 57 | 1.09 (0.85–1.39) | 202 | 1.29 (1.13–1.47)* | 0.18 |
| Sjögren’s disease | 152 | 1.20 (1.04–1.40) | 430 | 1.19 (1.09–1.30)* | 0.48 |
| Vitiligo | 20 | 1.32 (0.86–2.01) | 86 | 1.13 (0.93–1.38) | 0.25 |
| Immune thrombocytopenic purpura | 139 | 1.38 (1.17–1.62)* | 310 | 1.13 (1.02–1.25) | 0.04 |
| Pernicious anemia | 283 | 1.14 (1.02–1.27) | 530 | 1.16 (1.07–1.25)* | 0.69 |
| Psoriatic arthritis | 61 | 1.15 (0.91–1.46) | 289 | 1.18 (1.05–1.31) | 0.34 |
| Crohn's disease | 147 | 1.25 (1.08–1.46) | 547 | 1.13 (1.05–1.23) | 0.43 |
| Discoid lupus erythematosus | 37 | 1.26 (0.93–1.71) | 94 | 1.10 (0.91–1.33) | 0.55 |
| Guillain-Barré syndrome | 42 | 1.11 (0.84–1.48) | 119 | 1.20 (1.02–1.42) | 0.42 |
| Psoriasis | 517 | 1.14 (1.05–1.24)* | 1,523 | 1.15 (1.10–1.20)* | 0.52 |
| Celiac disease | 220 | 1.08 (0.95–1.22) | 883 | 1.16 (1.09–1.23)* | 0.65 |
| Systemic sclerosis | 37 | 1.05 (0.77–1.43) | 106 | 1.16 (0.97–1.37) | 0.28 |
| Ulcerative colitis | 345 | 1.18 (1.07–1.30)* | 1,214 | 1.09 (1.04–1.15)* | 0.14 |
| Ankylosing spondylitis | 86 | 1.19 (0.97–1.45) | 239 | 1.08 (0.96–1.21) | 0.10 |
| Rheumatoid arthritis | 1,220 | 1.13 (1.08–1.19)* | 2,773 | 1.09 (1.06–1.13)* | 0.44 |
| Lichen planus | 131 | 1.15 (0.98–1.35) | 437 | 1.08 (0.99–1.18) | 0.98 |
| Type I diabetes mellitus | 495 | 1.09 (1.00–1.18) | 1,257 | 1.08 (1.03–1.14) | 0.34 |
| Rheumatic fever/rheumatic heart diseases | 2,783 | 1.08 (1.04–1.12)* | 3,756 | 1.07 (1.04–1.10)* | 0.26 |
| Multiple sclerosis | 55 | 1.23 (0.96–1.58) | 289 | 1.06 (0.96–1.18) | 0.09 |
| Primary sclerosing cholangitis | 368 | 1.09 (1.00–1.20) | 721 | 1.05 (0.98–1.12) | 0.30 |
| Vasculitis | 461 | 1.06 (0.98–1.16) | 918 | 1.05 (0.99–1.12) | 0.77 |
| Polymyalgia rheumatica | 1,200 | 1.05 (1.00–1.11) | 1,315 | 1.02 (0.97–1.07) | 0.97 |
| Addison's disease | 85 | 1.35 (1.10–1.66) | 336 | 0.99 (0.90–1.09) | 0.01 |
| Autoimmune hemolytic anemia | 28 | 1.23 (0.86–1.76) | 81 | 1.00 (0.82–1.22) | 0.40 |
| Reactive arthritis | 4 | 1.03 (0.39–2.67) | 13 | 1.06 (0.63–1.78) | 0.90 |
| Graves' disease | 550 | 1.05 (0.98–1.14) | 1,526 | 1.01 (0.97–1.06) | 0.75 |
| Rheumatism, unspecified | 33 | 1.33 (0.97–1.82) | 210 | 1.00 (0.89–1.14) | 0.06 |
| Bullous disorders | 98 | 1.01 (0.84–1.22) | 137 | 1.01 (0.86–1.17) | 0.83 |
| Uveitis | 89 | 1.05 (0.86–1.28) | 232 | 1.00 (0.88–1.12) | 0.82 |
| Aplastic anemia | 280 | 1.01 (0.90–1.12) | 500 | 0.93 (0.86–1.01) | 0.92 |
| Dermato/polymyositis | 16 | 0.56 (0.32–0.99) | 50 | 1.04 (0.80–1.35) | 0.06 |
| Autoimmune thyroiditis | 32 | 0.84 (0.60–1.17) | 161 | 0.94 (0.81–1.08) | 0.47 |
| Primary biliary cholangitis | 34 | 0.89 (0.64–1.22) | 141 | 0.86 (0.74–1.00) | 0.75 |
| Pure red cell aplasia | 5 | 0.48 (0.19–1.19) | 6 | 0.46 (0.18–1.14) | 0.80 |

**Abbreviations**: IR, incidence rate; HR, hazard ratio; CI, confidence interval

*Indicates statistically significant after applying Bonferroni correction.

# **Supplementary Table 4.** Association between neutrophil-to-lymphocyte ratio and autoimmune diseases by sex

| **Autoimmune diseases** | **Female** | | **Male** | | **p-for-interaction** |
| --- | --- | --- | --- | --- | --- |
|  | **No of events** | **HR (95% CI)** | **No of events** | **HR (95% CI)** |  |
| Mixed connective tissue disease | 30 | 1.29 (0.92–1.81) | 5 | N/A | 0.99 |
| Sarcoidosis | 202 | 1.53 (1.34–1.75)* | 222 | 1.49 (1.31–1.70)* | 0.52 |
| Antiphospholipid syndrome | 100 | 1.42 (1.17–1.72)* | 64 | 1.43 (1.13–1.81) | 0.54 |
| Autoimmune hepatitis | 161 | 1.35 (1.16–1.56)* | 41 | 1.48 (1.09–2.02) | 0.60 |
| Scleroderma (localized) | 25 | 1.44 (0.99–2.10) | 13 | 1.44 (0.85–2.42) | 0.94 |
| Systemic lupus erythematosus | 214 | 1.29 (1.14–1.46)* | 45 | 1.24 (0.94–1.64) | 0.97 |
| Sjögren’s disease | 523 | 1.15 (1.06–1.25)* | 59 | 1.45 (1.13–1.85) | 0.13 |
| Vitiligo | 62 | 1.08 (0.86–1.37) | 44 | 1.23 (0.93–1.63) | 0.74 |
| Immune thrombocytopenic purpura | 211 | 1.20 (1.06–1.36) | 238 | 1.21 (1.07–1.37) | 0.92 |
| Pernicious anemia | 514 | 1.18 (1.09–1.28)* | 299 | 1.12 (1.01–1.25) | 0.35 |
| Psoriatic arthritis | 188 | 1.19 (1.04–1.36) | 162 | 1.13 (0.98–1.31) | 0.26 |
| Crohn's disease | 376 | 1.10 (1.00–1.21) | 318 | 1.25 (1.12–1.38)* | 0.20 |
| Discoid lupus erythematosus | 100 | 1.25 (1.04–1.50) | 31 | 1.03 (0.74–1.43) | 0.19 |
| Guillain-Barré syndrome | 61 | 1.12 (0.89–1.42) | 100 | 1.20 (1.00–1.44) | 0.74 |
| Psoriasis | 979 | 1.18 (1.11–1.25)* | 1,061 | 1.11 (1.05–1.17)* | 0.26 |
| Celiac disease | 659 | 1.13 (1.05–1.21)* | 444 | 1.16 (1.06–1.26) | 0.67 |
| Systemic sclerosis | 115 | 1.18 (0.99–1.40) | 28 | 0.93 (0.67–1.31) | 0.31 |
| Ulcerative colitis | 731 | 1.12 (1.05–1.20)* | 828 | 1.11 (1.05–1.18)* | 0.55 |
| Ankylosing spondylitis | 147 | 1.00 (0.86–1.16) | 178 | 1.24 (1.08–1.43) | 0.03 |
| Rheumatoid arthritis | 2,626 | 1.10 (1.07–1.14)* | 1,367 | 1.12 (1.06–1.17)* | 0.60 |
| Lichen planus | 397 | 1.11 (1.02–1.22) | 171 | 1.07 (0.93–1.23) | 0.40 |
| Type I diabetes mellitus | 713 | 1.09 (1.02–1.16) | 1,039 | 1.11 (1.05–1.17)* | 0.52 |
| Rheumatic fever/rheumatic heart diseases | 2,791 | 1.08 (1.04–1.11)* | 3,748 | 1.08 (1.05–1.11)* | 0.65 |
| Multiple sclerosis | 226 | 1.07 (0.95–1.21) | 118 | 1.15 (0.97–1.35) | 0.32 |
| Primary sclerosing cholangitis | 469 | 1.03 (0.95–1.12) | 620 | 1.08 (1.01–1.16) | 0.69 |
| Vasculitis | 817 | 1.08 (1.01–1.15) | 562 | 1.08 (1.00–1.17) | 0.55 |
| Polymyalgia rheumatica | 1,541 | 1.04 (0.99–1.09) | 974 | 1.09 (1.03–1.16) | 0.45 |
| Addison's disease | 220 | 1.00 (0.89–1.12) | 201 | 1.11 (0.98–1.26) | 0.53 |
| Autoimmune hemolytic anemia | 47 | 1.33 (1.02–1.74) | 62 | 0.96 (0.76–1.20) | 0.10 |
| Reactive arthritis | 3 | 0.85 (0.29–2.53) | 14 | 1.06 (0.64–1.74) | 0.96 |
| Graves' disease | 1,569 | 1.03 (0.98–1.08) | 507 | 1.05 (0.97–1.14) | 0.79 |
| Rheumatism, unspecified | 195 | 0.99 (0.87–1.13) | 48 | 1.23 (0.94–1.60) | 0.08 |
| Bullous disorders | 99 | 1.08 (0.90–1.29) | 136 | 0.98 (0.84–1.14) | 0.31 |
| Uveitis | 186 | 1.04 (0.90–1.19) | 135 | 0.95 (0.81–1.12) | 0.96 |
| Aplastic anemia | 290 | 1.04 (0.93–1.15) | 490 | 0.92 (0.85–1.00) | 0.37 |
| Dermato/polymyositis | 52 | 0.92 (0.71–1.19) | 14 | 1.08 (0.66–1.76) | 0.39 |
| Autoimmune thyroiditis | 173 | 0.89 (0.77–1.02) | 20 | 0.93 (0.61–1.42) | 0.93 |
| Primary biliary cholangitis | 141 | 0.86 (0.74–1.01) | 34 | 0.90 (0.67–1.22) | 0.90 |
| Pure red cell aplasia | 5 | 0.32 (0.09–1.15) | 6 | 0.45 (0.19–1.04) | 0.60 |

**Abbreviations**: IR, incidence rate; HR, hazard ratio; CI, confidence interval

*Indicates statistically significant after applying Bonferroni correction.

# **Supplementary Table 5.** Sensitivity analyses results on the association between neutrophil-to-lymphocyte ratio and autoimmune diseases

|  | **Lag time of 5-year** | | **Excluding immunocompromised** | |
| --- | --- | --- | --- | --- |
| **Autoimmune diseases** | **No of events** | **HR (95% CI)** | **No of events** | **HR (95% CI)** |
| Mixed connective tissue disease | 18 | 1.19 (0.78–1.81) | 33 | 1.49 (1.08–2.07) |
| Sarcoidosis | 332 | 1.42 (1.27–1.57)* | 382 | 1.52 (1.38–1.68)* |
| Antiphospholipid syndrome | 140 | 1.42 (1.21–1.67)* | 155 | 1.44 (1.24–1.68)* |
| Autoimmune hepatitis | 165 | 1.37 (1.18–1.59)* | 184 | 1.34 (1.17–1.54)* |
| Scleroderma (localized) | 23 | 1.25 (0.86–1.83) | 32 | 1.37 (0.99–1.91) |
| Systemic lupus erythematosus | 207 | 1.18 (1.04–1.34) | 233 | 1.21 (1.07–1.37) |
| Sjögren’s disease | 458 | 1.13 (1.03–1.23) | 518 | 1.20 (1.10–1.30)* |
| Vitiligo | 91 | 1.21 (0.99–1.46) | 98 | 1.23 (1.02–1.49) |
| Immune thrombocytopenic purpura | 350 | 1.22 (1.10–1.34)* | 383 | 1.16 (1.05–1.28) |
| Pernicious anemia | 589 | 1.18 (1.10–1.28)* | 730 | 1.17 (1.10–1.26)* |
| Psoriatic arthritis | 287 | 1.18 (1.06–1.32) | 327 | 1.15 (1.04–1.27) |
| Crohn's disease | 542 | 1.14 (1.05–1.23)* | 611 | 1.14 (1.06–1.23)* |
| Discoid lupus erythematosus | 96 | 1.17 (0.97–1.41) | 115 | 1.14 (0.96–1.36) |
| Guillain-Barré syndrome | 114 | 1.12 (0.94–1.33) | 142 | 1.19 (1.02–1.39) |
| Psoriasis | 1,748 | 1.16 (1.11–1.21)* | 1,840 | 1.15 (1.10–1.20)* |
| Celiac disease | 826 | 1.12 (1.05–1.20)* | 1,009 | 1.14 (1.07–1.21)* |
| Systemic sclerosis | 113 | 1.15 (0.97–1.37) | 126 | 1.13 (0.96–1.33) |
| Ulcerative colitis | 1,173 | 1.13 (1.07–1.19)* | 1,389 | 1.12 (1.06–1.17)* |
| Ankylosing spondylitis | 267 | 1.16 (1.03–1.30) | 289 | 1.13 (1.01–1.26) |
| Rheumatoid arthritis | 3,269 | 1.10 (1.06–1.13)* | 3,547 | 1.12 (1.09–1.16)* |
| Lichen planus | 440 | 1.07 (0.98–1.17) | 513 | 1.09 (1.00–1.18) |
| Type I diabetes mellitus | 1,227 | 1.12 (1.06–1.18)* | 1,545 | 1.08 (1.03–1.13)* |
| Rheumatic fever/rheumatic heart diseases | 5,770 | 1.07 (1.04–1.09)* | 5,752 | 1.07 (1.04–1.09)* |
| Multiple sclerosis | 255 | 1.06 (0.95–1.19) | 308 | 1.07 (0.97–1.19) |
| Primary sclerosing cholangitis | 944 | 1.09 (1.03–1.16) | 952 | 1.05 (0.99–1.11) |
| Vasculitis | 1,103 | 1.06 (1.00–1.11) | 1,203 | 1.07 (1.02–1.13) |
| Polymyalgia rheumatica | 2,183 | 1.06 (1.02–1.10) | 2,227 | 1.07 (1.03–1.11)* |
| Addison's disease | 361 | 1.02 (0.93–1.13) | 340 | 1.06 (0.96–1.17) |
| Autoimmune hemolytic anemia | 90 | 1.03 (0.85–1.24) | 95 | 0.95 (0.79–1.14) |
| Reactive arthritis | 11 | 0.81 (0.46–1.44) | 15 | 1.22 (0.75–1.99) |
| Graves' disease | 1,630 | 1.04 (1.00–1.09) | 1,836 | 1.03 (0.99–1.08) |
| Rheumatism, unspecified | 38 | 1.29 (0.95–1.75) | 220 | 1.05 (0.93–1.19) |
| Bullous disorders | 183 | 1.03 (0.90–1.18) | 198 | 1.02 (0.89–1.16) |
| Uveitis | 271 | 0.99 (0.88–1.11) | 290 | 1.01 (0.91–1.13) |
| Aplastic anemia | 635 | 0.99 (0.92–1.06) | 634 | 0.96 (0.90–1.04) |
| Dermato/polymyositis | 53 | 0.88 (0.68–1.14) | 62 | 1.01 (0.80–1.28) |
| Autoimmune thyroiditis | 150 | 0.86 (0.74–1.00) | 166 | 0.94 (0.81–1.09) |
| Primary biliary cholangitis | 130 | 0.90 (0.76–1.06) | 152 | 0.86 (0.74–1.00) |
| Pure red cell aplasia | 5 | 0.35 (0.13–0.99) | 7 | 0.29 (0.10–0.81) |

**Abbreviations**: IR, incidence rate; HR, hazard ratio; CI, confidence interval

*Indicates statistically significant after applying Bonferroni correction.

# **Supplementary Table 6.** Association between neutrophil-to-lymphocyte ratio and autoimmune diseases across quartiles

| **Autoimmune diseases** | **Q2 vs. Q1 HR (95% CI)** | **Q3 vs. Q1**  **HR (95% CI)** | **Q4 vs. Q1**  **HR (95% CI)** |
| --- | --- | --- | --- |
| Mixed connective tissue disease | 0.40 (0.11–1.53) | 0.96 (0.34–2.68) | 2.53 (1.07–5.98) |
| Sarcoidosis | 1.57 (1.14–2.18) | 1.69 (1.22–2.35) | 3.61 (2.67–4.87)* |
| Antiphospholipid syndrome | 1.06 (0.63–1.77) | 1.65 (1.02–2.66) | 2.83 (1.79–4.47)* |
| Autoimmune hepatitis | 1.09 (0.70–1.69) | 1.28 (0.83–1.97) | 2.47 (1.65–3.68)* |
| Scleroderma (localized) | 0.42 (0.11–1.64) | 2.51 (1.04–6.07) | 1.65 (0.63–4.31) |
| Systemic lupus erythematosus | 0.88 (0.61–1.27) | 1.15 (0.81–1.64) | 1.85 (1.31–2.60)* |
| Sjögren’s disease | 1.25 (0.99–1.58) | 1.05 (0.82–1.35) | 1.93 (1.53–2.44)* |
| Vitiligo | 1.20 (0.69–2.10) | 1.13 (0.63–2.03) | 1.85 (1.06–3.21) |
| Immune thrombocytopenic purpura | 0.95 (0.71–1.28) | 1.05 (0.79–1.40) | 1.65 (1.27–2.15)* |
| Pernicious anemia | 1.17 (0.95–1.45) | 1.28 (1.03–1.57) | 1.64 (1.34–2.01)* |
| Psoriatic arthritis | 1.19 (0.85–1.65) | 1.62 (1.18–2.21) | 1.53 (1.10–2.11) |
| Crohn's disease | 1.33 (1.06–1.66) | 1.05 (0.83–1.33) | 1.71 (1.37–2.12)* |
| Discoid lupus erythematosus | 0.56 (0.32–0.95) | 0.82 (0.50–1.35) | 1.45 (0.92–2.30) |
| Guillain-Barré syndrome | 0.97 (0.60–1.58) | 1.20 (0.75–1.90) | 1.46 (0.94–2.28) |
| Psoriasis | 1.22 (1.07–1.40) | 1.38 (1.21–1.57)* | 1.54 (1.35–1.75)* |
| Celiac disease | 1.05 (0.88–1.26) | 1.21 (1.02–1.44) | 1.46 (1.23–1.74)* |
| Systemic sclerosis | 1.25 (0.78–2.01) | 1.25 (0.77–2.04) | 1.50 (0.92–2.44) |
| Ulcerative colitis | 1.27 (1.09–1.48) | 1.32 (1.14–1.54)* | 1.49 (1.28–1.72)* |
| Ankylosing spondylitis | 0.94 (0.68–1.32) | 1.11 (0.80–1.53) | 1.36 (0.99–1.86) |
| Rheumatoid arthritis | 1.04 (0.95–1.14) | 1.14 (1.04–1.25) | 1.39 (1.27–1.52)* |
| Lichen planus | 0.95 (0.74–1.21) | 1.19 (0.94–1.50) | 1.29 (1.02–1.64) |
| Type I diabetes mellitus | 0.96 (0.83–1.11) | 1.03 (0.89–1.19) | 1.25 (1.09–1.43)* |
| Rheumatic fever/rheumatic heart diseases | 1.06 (0.99–1.15) | 1.05 (0.98–1.13) | 1.27 (1.18–1.36)* |
| Multiple sclerosis | 1.13 (0.83–1.55) | 1.17 (0.86–1.60) | 1.26 (0.93–1.71) |
| Primary sclerosing cholangitis | 1.13 (0.95–1.36) | 1.09 (0.91–1.30) | 1.28 (1.07–1.52) |
| Vasculitis | 1.02 (0.88–1.20) | 1.04 (0.89–1.21) | 1.23 (1.06–1.43) |
| Polymyalgia rheumatica | 1.05 (0.93–1.17) | 1.12 (1.00–1.26) | 1.18 (1.05–1.32) |
| Addison's disease | 1.11 (0.84–1.46) | 0.80 (0.60–1.08) | 1.25 (0.96–1.63) |
| Autoimmune hemolytic anemia | 0.70 (0.40–1.22) | 0.57 (0.32–1.02) | 1.10 (0.68–1.79) |
| Reactive arthritis | 1.36 (0.36–5.21) | 0.80 (0.17–3.73) | 1.31 (0.32–5.37) |
| Graves' disease | 1.10 (0.97–1.24) | 1.07 (0.95–1.22) | 1.13 (0.99–1.28) |
| Rheumatism, unspecified | 1.34 (0.94–1.91) | 0.95 (0.65–1.40) | 1.25 (0.86–1.80) |
| Bullous disorders | 0.85 (0.59–1.24) | 0.62 (0.42–0.93) | 1.11 (0.79–1.57) |
| Uveitis | 1.16 (0.85–1.57) | 0.89 (0.64–1.24) | 1.15 (0.83–1.59) |
| Aplastic anemia | 0.77 (0.62–0.94) | 0.76 (0.62–0.93) | 0.86 (0.70–1.04) |
| Dermato/polymyositis | 1.29 (0.71–2.37) | 0.43 (0.18–1.03) | 1.18 (0.59–2.36) |
| Autoimmune thyroiditis | 0.96 (0.67–1.39) | 0.72 (0.48–1.09) | 0.84 (0.56–1.28) |
| Primary biliary cholangitis | 0.75 (0.51–1.12) | 0.75 (0.50–1.12) | 0.64 (0.42–1.00) |
| Pure red cell aplasia | 0.28 (0.06–1.39) | 0.13 (0.02–1.07) | 0.10 (0.01–0.83) |

**Abbreviations**: IR, incidence rate; HR, hazard ratio; CI, confidence interval

*Indicates statistically significant after applying Bonferroni correction.


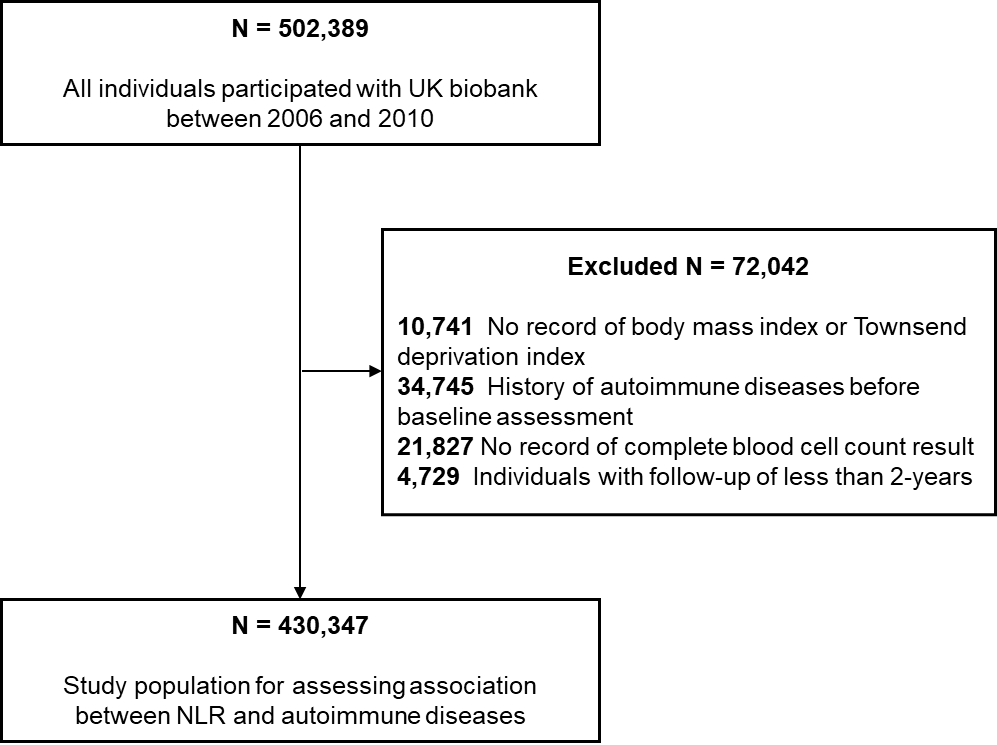


**Supplementary Figure 1.** Flow chart of population selection criteria in the UK biobank database

*Notes: Exclusion were performed in the order presented in this figure.*


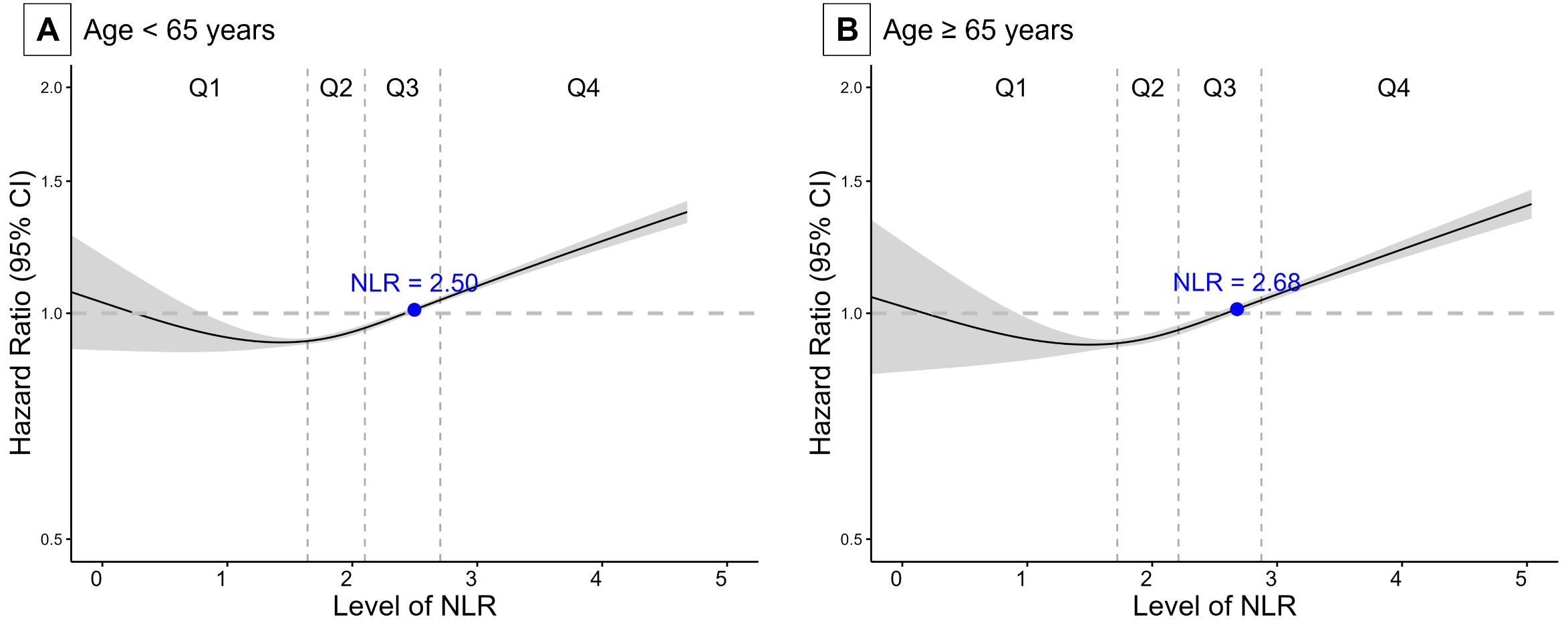


**Supplementary Figure 2.** Spline curve of neutrophil-to-lymphocyte ratio and risk of autoimmune diseases by age group

Notes: The NLR value of 2.50 or 2.68 with blue dot represents the point where the lower confidence interval of the hazard ratio exceeds 1.
